# Investigating Prebiotic Protocells for a Comprehensive Understanding of the Origins of Life: A Prebiotic Systems Chemistry Perspective †

**DOI:** 10.3390/life9020049

**Published:** 2019-06-07

**Authors:** Augustin Lopez, Michele Fiore

**Affiliations:** 1 Institut de Chimie et Biochimie Moléculaires et Supramoléculaires, Université de Lyon, Claude Bernard Lyon 1, 1 Rue Victor Grignard, Bâtiment Lederer, 69622 Villeurbanne CEDEX, France; augustin.lopez@ens-lyon.fr; 2 Master de Biologie, École Normale Supérieure de Lyon, Université Claude Bernard Lyon I, Université de Lyon, 69342 Lyon CEDEX 07, France

**Keywords:** protocell, compartment, prebiotic chemistry, systems chemistry, origin of life

## Abstract

Protocells are supramolecular systems commonly used for numerous applications, such as the formation of self-evolvable systems, in systems chemistry and synthetic biology. Certain types of protocells imitate plausible prebiotic compartments, such as giant vesicles, that are formed with the hydration of thin films of amphiphiles. These constructs can be studied to address the emergence of life from a non-living chemical network. They are useful tools since they offer the possibility to understand the mechanisms underlying any living cellular system: Its formation, its metabolism, its replication and its evolution. Protocells allow the investigation of the synergies occurring in a web of chemical compounds. This cooperation can explain the transition between chemical (inanimate) and biological systems (living) due to the discoveries of emerging properties. The aim of this review is to provide an overview of relevant concept in prebiotic protocell research.

## 1. Introduction: Chemical Basis for the Emergence of Protocells

Today, despite the enormous scientific achievements reached in the field of the applied science, such as medicine, biology, engineering, physics and chemistry, many interrogative points accompany the life of scientists. One of humanity’s most asked questions is how and where did we come from, and in a broader sense, how life emerged on our planet and perhaps, in the universe [1]. In order to unravel the mystery, collaborations between many disciplines of research are performed (chemistry, biology, geology, etc.). As life must have emerged from prebiotic chemistry [2], prior to developing its own chemistry called biochemistry [3], the former discipline is noteworthy to investigate how life appeared from an inanimate mixture of chemical compounds.

A small number of common molecules is at the basis of the cells, which are the starting point of life [4]. This similarity tends to show that lipids, nucleotides and amino acids are mandatory for the outbreak of life. Their sources are then a major topic in the study of the origins of life. The question of how and where these compounds were synthetized in abiotic conditions fascinated scientist in the last 60 years and is still an open question.

These building blocks of life could have either come from space by meteorites or they could have already been present on the primitive Earth [5]. Amphiphilic compounds, nucleotides and amino acids, were found in different amounts in carbonaceous meteorites [6] and glycine (the simplest amino acid) is present in very small parts in interstellar dust [7]. Research in prebiotic chemistry has given several plausible explanations for the formation of important biomolecules, such as amino acids and nucleotides, and different chemical approaches have been considered.

**Figure 1:**
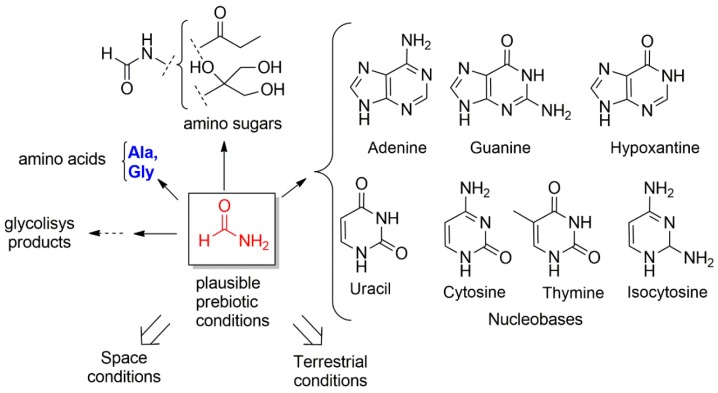
The formamide clue studied during the last 20 years by Saladino and colleagues. Amino acids (in blue), nucleobases and lipid precursors (not shown) can notably be produced by combination of formamide (in red) and other small carbon based molecules.

**Figure 2:**
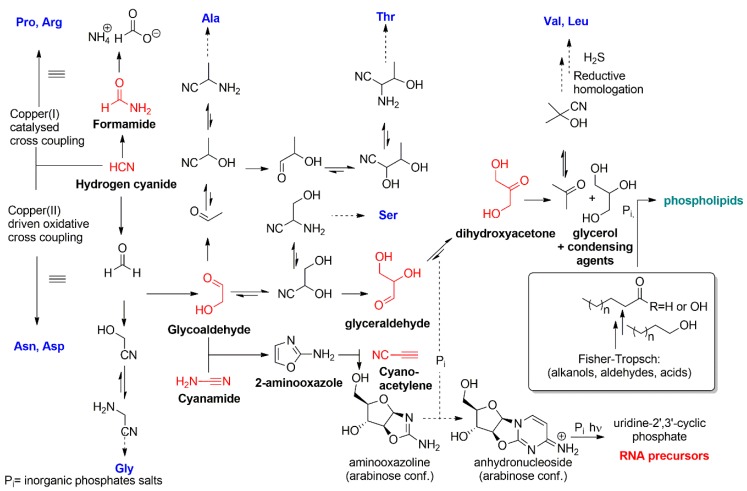
A summary of some plausible prebiotic routes for the formation of aminoacids, nucleic acids and phospholipids from common building blocks. Prebiotic synthesis of phospholipids and other amhiphiles was summarized elsewhere [24].

A significant boost was given by the pioneering—and prebiotic systems chemistry—experiments of Stanley Miller, later revisited by Jeffrey Bada and colleagues. These experiments showed that in some prebiotic conditions, such as, spark discharges [8,9], volcanic spark discharges [10], and H_2_S enriched discharges [11], a soup containing small molecules in the presence of other gas molecules, can generate a large variety of proteogenic and non proteogenic aminoacids (Table 1). For Raffaele Saladino and colleagues, formamide is the chemical clue at the origin of relevant prebiotic compounds, such as nucleic acids and some aminoacids [12] (Figure 2). The term, formamide clue, is due to the fact that formamide (Figure 2), in combination with small molecules, such as ammonia (NH_3_) and cyanuric acid (HCN) in acidic conditions, could be a universal building block for the abiotic syntheses of biotic precursors. Raffaele Saladino and colleagues explored in the last two decades any possible prebiotic plausible conditions, including the irradiation of formamide with UV light or a proton beam (simulating the Solar wind) in the presence of different meteorite powders (Murchinson’s fragments) and minerals (Borate, Titanium oxides, FeS, Phosphates). Several molecules (Figure 1) were found in the formamide based prebiotic mixture, and among others, polyhydroxy alcohols and carboxylic acids (amphiphilic compounds), amino acids (Ala and Gly), dehydrating agents such as urea, nitriles and *N*-heterocycles (RNA precursors) [13,14].

Another relevant research study consisted in simulating the synthesis of nucleobases (Figure 2) in plausible prebiotic conditions [15,16]. Pioneering works were carried out by Oro and colleagues that obtained some nucleotides from a mixtures of HCN, CH_4_, NH_3_ and H_2_O [17]. Zipse, Carell and colleagues obtained several purine nucleosides from the double cyclization of formamido pyrimidines in the presence of ribose or lower sugars under aqueous conditions [18]. Lately, Powner and colleagues demonstrated how pyrimidine nucleotides and 8-oxopurine nucleotides could be formed under similar prebiotic conditions [19]. Steve Benner and colleagues have also found alternative plausible prebiotic pathways for the synthesis of Nicotinamide ribose 2’-phosphate [20].

Although the current data explain only partially the abiotic synthesis of nucleosides, amino acids and amphiphiles, a major step has been achieved in the recent years with the work carried out by John Sutherland and colleagues. They have shown that several important classes of biomolecules, such as 2’,3’-cyclic pyrimidine nucleotides, various–amino acids and glycerol phosphate, may have hydrogen cyanide and formaldehyde as common chemical precursors in a so called cyanosulfidic proto-metabolism (Figure 2) [21,22,23].

At some point, the molecules needed for life became compartmentalized with an internal volume separated from an external volume by a boundary [25]. Compartmentalization it is an important step because this boundary gives numerous advantages for a chemical system. In terms of metabolism, a boundary represents a barrier separating an inner medium from an external medium. Thus, the concentration of the reactants can be higher inside the compartment and then the rate of the catalyzes is increased. The difference of conditions between each side of the limit can be controlled by selective permeability and regulated exchanges. These mechanisms ensure the supply of the system and the exit of the waste. Otherwise, a boundary is a privileged interface for the formation of gradients which could be exploited as an energetic source [25]. Moreover, the fact of having a system separated from the medium allows the existence and the maintenance of its own identity defined by its composition. If synergies emerge between the different components, the fitness, defined here as the replication potential of the system, can be enhanced. Therefore, this system would thrive compared to others if they compete with one another. On the other hand, a protocell containing parasites would have a lower fitness, explaining its defeat in the competition [26].

**Figure 3:**
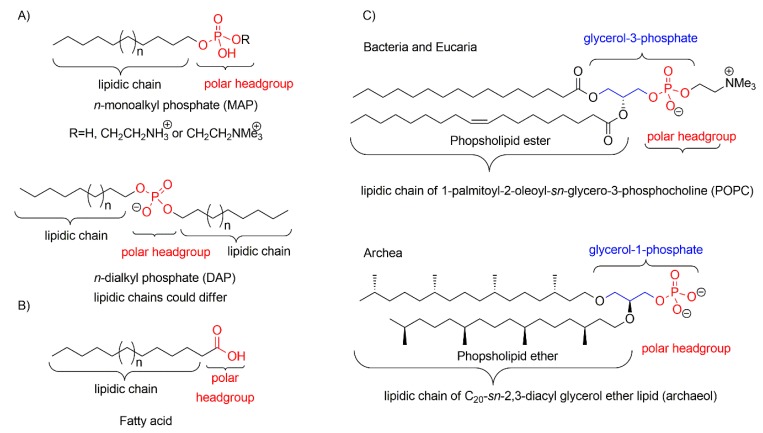
The structure of some amphiphiles that have been studied as first protocells membrane constituents: (**A**) Different mono- and dialkyl phosphates structures; (**B**) a general structure of a fatty acid; (**C**) The structure of ester and ether phospholipids.

In modern cells these boundaries (Box 2) are made of phospholipids, cholesterol and phospholipid ethers typically found in *archea* species [27] (Figure 3). The prebiotic synthesis of complete lipids, such as phosphatidylate, phosphoethanolamine, phosphatidylcholine and glycerophosphate, [24] was firstly explored by Deamer and colleagues [28] and further completed by Oro and colleagues [29,30,31,32]. As a general scheme, those syntheses were carried out by concomitant condensation of glycerol, phosphate source (Pi) and fatty acids, alcohols or aldehydes (source of the lipid chains) yielding small amounts of complete lipids (0.015% to 0.45%). The condensing agents used include urea or cyanamide, and phospholipids were obtained at moderate temperatures (60–100 °C) and in variable reaction times (7–96 h). It is noteworthy that phospholipid precursors, such as glycerol-phosphate, or 2-aminoethyl phosphate, were obtained in similar prebiotic conditions [24]. In addition, one of the authors recently showed that some phospholipids can be also be obtained by a direct phosphorylation of mono- and di-acyl glycerols [33].

**Figure 4:**
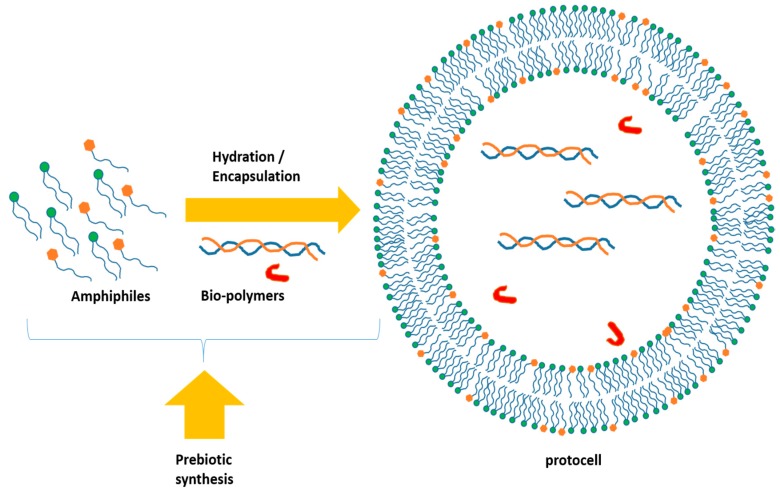
The spontaneous appearance of closed membranes composed of bilayers of self-assembling amphiphiles was likely a prerequisite for Darwinian competitive behavior to set in at the molecular level. Such compartments should be dynamic in their membrane composition (evolvable), sufficiently stable to harbor macro-molecules, yet semi-permeable for reactive small molecules to get across the membrane and the content to be able to avoid chemical equilibrium.

However, due to the complex structures of phospholipids and complete lipids, it is plausible that prebiotic compartments were made of long chain fatty molecules such as fatty acids, long chain alcohols, monoalkyl and dialkyl phosphates [34]. Indeed, the hydration of a crude prebiotic mixture of some of these amphiphilic compounds formed membrane bilayers and multilamellar giant vesicles under the good hydration conditions (pH, temperature, salt concentrations, presence of biopolymers) and the presence of plausibly prebiotic co-surfactants [35]. Such compartments may be able to encapsulate biomolecules, such as short peptide chains, DNA/RNA or ribozymes strands as well as enzymes, that can trigger a sort of primitive pathway in order to constitute a plausible prebiotic compartment (Figure 4).

The prebiotic preparation of some of these amphiphiles, as well as nucleotides and aminoacids, have been studied by different research groups. Among others, the first study of Deamer and colleagues [36] prompted not only an investigation of the prebiotic synthesis of phospholipids [24,28,29,30,31,32], but also the preparation of mono-alkyl phosphates with even [37,38] and odd numbers [35] of carbon atoms on the long fatty chain. It was also proven that the condensation of small molecules into biopolymers and the phosphorylation of primary and secondary alcohols is related to the presence of highly energetic molecules that act as condensing agents [24].

**Figure 5:**
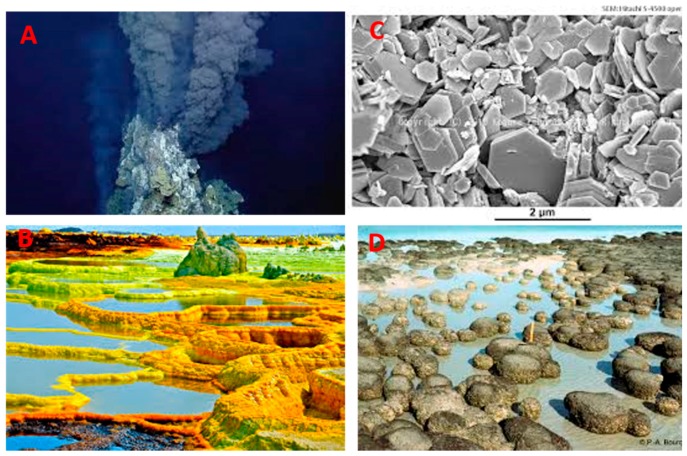
(**A**) Hydrothermal vents on the bottom of Atlantic ocean; (**B**) A hydrothermal pool in Dallo (Africa); (**C**) Electron microscopic imagine of a clay mineral; (**D**) Stromatolite in western Australia. Public domain images.

Plausible prebiotic scenarios for the synthesis of previously mentioned molecules are different. However, modern scientists, in the impossibility to execute time travel, depicted different (and plausible) geochemical scenarios for the prebiotic synthesis of aminoacids, nucleotides and phospholipids or phospholipids precursors. The plausibly scenarios have a common leitmotiv, that is, the presence of liquid water, a source of energy (geothermic but also sun-light/ UV radiation), and the presence of minerals and organic carbon. These conditions are reunited in hydrothermal vents and hydrothermal fields which were largely represented on the young Earth. For instance, it is possible that long chain alcohols together with fatty acids synthesized in these environments where beads of minerals should catalyze the formation of H–C, C–C and C–O bonds at reasonably high temperatures (>300 ° C) in the Fischer-Tropsch reaction type [39,40]. Hydrothermal vents are present today at the bottom of the oceans (Figure 5A). They are systems whose heat source is the underlying magma or hot water generated by convection currents due to high thermal gradients [41]. Hydrothermal vents, also called hydrothermal black smokers or submarine hot springs, are alkaline, and far from equilibrium environments [42]. Their discoveries have been proposed as sites at which chemical reactions could initiate a primitive metabolism involving the reduction of CO_2_ by dissolved H_2_ [43]. The alternative scenario is represented by the hydrothermal fields (Figure 5B), which are also known as hydrothermal pools or geysers. Hydrothermal vents are low pH range reactors and can have as energy sources, not only naturally occurring geothermy, but also can be subject to UV irradiations. In recent years, Damer and Deamer pointed out that hydrothermal pools could be considered as plausibly prebiotic reactors for the synthesis and the polymerization of several key molecules in the development of life, including lipids, nucleic acids and peptides. One crucial feature is the fluctuation of hydrated and dehydrated conditions related to precipitation and evaporation of water on volcanic land masses [44]. In other words, geysers could be the receptacles of organic, moderately hydrophobic compounds that precipitated, then fell into a hydrothermal field and accumulated, like a bathtub ring, around its borders at the fluctuating water-atmosphere interface. 

It is very important to highlight that today scientific evidences showed that, not only the presence of an hydrothermal source is necessary for promoting certain chemical reactions, but also the presence of some minerals, such as clays (Figure 5C). This is due to the fact that they can offer a better surface contact for the prebiotic synthesis of such molecules [45]. It is likely that the prebiotic formation of the first membrane-forming amphiphiles are able to encapsulate a primitive genetic code or a catalytic protein which occurred and coincided with the appearance of prebiotic amino acids (Table 1) and *N*-heterocycles. They could all be produced from the available geochemical sources, and also by the infusion of extraterrestrial material and gave rise to the formation of the first protocells that evolved into the first microorganisms. In fact, stromatolites (Figure 5D)—layered mounds, columns, and sheet-like sedimentary rocks that were originally formed by the growth with layer upon layer of cyanobacteria, a single-celled photosynthesizing microbe—represent the microfossil evidences that life started on our planet at least 3.4 billion years ago [46].

In order to understand how life could have appeared on a primitive Earth, researchers try to reproduce an artificial form of life [2]. Artificial life can be described as a cellular system able to self-reproduce and subject to Darwinian evolution [47]. The privileged models to investigate artificial life and to understand the emergence of contemporary cells are protocells. Several chemical systems have been investigated in in synthetic biology [48] and which were initially designed to investigate the origins of life [49,50]. Indeed, these compartmentalized systems are convenient to understand the transition from chemistry to biology occurring during the emergence of life. Two main approaches exist (top-down or bottom-up) concerning the choice of the protocell studied depending on the question which is addressed (Box 1).

Box 1 **The seminal idea of Systems Chemistry and the bottom-up approach in the origins of life study.**

The main purpose of synthetic chemistry is the preparation of pure, designed compounds by using well-defined synthetic pathways and multi-step reactions with the purpose to increase the yields as much as possible. However, the paradigm of the pure compound has hindered the development of complex chemical systems and the investigation of networks of chemical reaction. The preparation of a complex mixture of chemical compounds can be highly interesting since simple mixtures of non-reacting molecules can trigger chemical reaction networks including feedback loops and elements of non-linearity form. From such systems, new, unexpected and unpredicted, emergent properties could arise while none of the components alone has these properties. Systems chemistry is therefore an extremely interesting and very new way to do - and to think and re-think – chemistry beyond the fundamental insight that we can get. Current science, including synthetic chemistry, uses a top-down approach in order to establish the causes of a phenomenon, or in case of synthetic chemistry, the preparation of a compound, by following well defined retrosynthetic pathways. Systems chemistry though can be interpreted as a bottom-up approach which has the aim to piece together the parts of a whole to give rise to a new and more complex system with emerging proprieties.

Concerning the study of protocellular systems, the top-down approach consists in simplifying the machinery of contemporary cells, to obtain a simple but efficient system which would resemble to the primitive cells and which could explain how they worked. However, the bottom-up approach investigates the emergence of a living system properties due to the cooperation between simple molecules as chemical mixture likely to be present in a prebiotic environment. This way of proceeding tends to reproduce the process which led to the outbreak of life and biology from chemistry [2].

**Figure 6:**
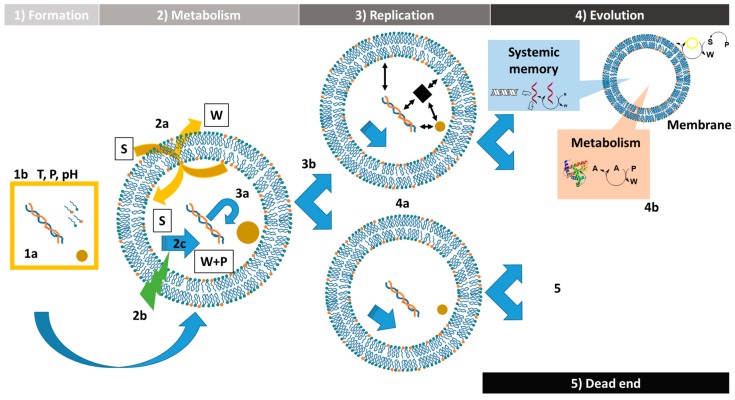
The life cycle and evolution of the protocells. 1: Formation. Protocells can emerge from a mixture of compounds (**1a**) which are present in a specific medium with characteristic conditions such as T: Temperature, P: Pressure, pH (**1b**). 2: Metabolism. Functional protocells are able to exchange with the medium (**2a**) and they possess a source of energy (**2b**) which fuels a catalytic network (**2c**) producing products (P) and waste (W) from substrates (S). 3: Replication. Protocell division involves the replication of some of its content (**3a**) but also the replication of the container (**3b**). 4: Evolution. Through divisions, protocells can acquire new compounds involving a higher fitness for the systems (**4a**). These protocells are preferentially conserved during selection and evolution (**4b**). On the other hand, if a protocell does not keep the different acquired networks or has less efficient networks, then it will lose the competition, and in that case it is a dead end for the system (**5**). This figure was adapted from Kee et al., 2017 [51].

This review underlines the usefulness of protocells to investigate the fundamental processes of the origins of any living system: Its formation, its functioning, its reproduction and its evolution (Figure 6). The importance of the interactions and the complexity of these chemical systems is also highlighted during the different steps towards the outbreak of life.

## 2. Formation of the Protocells

The first step for the emergence of life is the establishment of a chemical system from a mixture of compounds in characteristic conditions (Figure 6).

Several types of compartmentalization have been proposed in a prebiotic environment. Simple compartments such as the coacervates, which are membraneless compartments made of oppositely charged macromolecules and ions, are also commonly investigated as basic protocells [25]. However, the study of lipids for the formation of protocells remain the most studied model since the lipid boundary make them the closest system possible to modern cells [25] (Box 2). These vesicles could be generated spontaneously by self-assembly of amphiphilic molecules available in the medium. Interestingly, membranes made of mixtures of lipids, for instance, short chain fatty acids (<7 carbons) with medium chain fatty acids (8–12 carbons), or containing other types of molecules, were showed to be more stable, [52] and still relevant because they could all be present on the primitive Earth [5]. The vesiculation event explains how a protocell constituted by numerous types of compounds can be generated by entrapment and the membrane self-assembly [53]. Interestingly, macromolecules from the medium, such as ribosomes or even a mixture or proteins (the PURE system which is a commercial in vitro protein synthesis kit constituted by various enzymes), are naturally encapsulated in liposomes during their formations [54]. The first studies of vesicle formation were essentially based on one type of fatty acids, such as oleic acids, for biological relevance. As many factors can influence the protocell formation, this phenomenon should be considered in a local geochemical environment for the bottom-up approaches.

Box 2 **What is a giant vesicle? Why and how to prepare it?**

Giant vesicle are vesicles with a diameter greater than 1 µm. There is a considerable interest in preparing cell-sized (10–50 µm) giant (unilamellar or multilammellar) vesicles (GVs) from natural (and or) non-natural amphiphiles: the membranes of those compartments resemble to the modern cells ones. GVs can also include biopolymers with the ultimate goal of constructing a dynamic artificial cell-like system (Figure 4). To prepare GVs, a thin dried film of amphiphilic compounds, prepared by evaporation of chloroform or methanol in which the lipids were diluted, is hydrated by using an appropriate buffer. The hydration is performed for a minimum of 12h with a control of the temperature. After the hydration time a turbid solution containing GVs can be observed depending on the concentration of lipids. The film of amphiphilic compounds or the internal buffer can be enriched in fluorescent molecules to observe the vesicles formed.

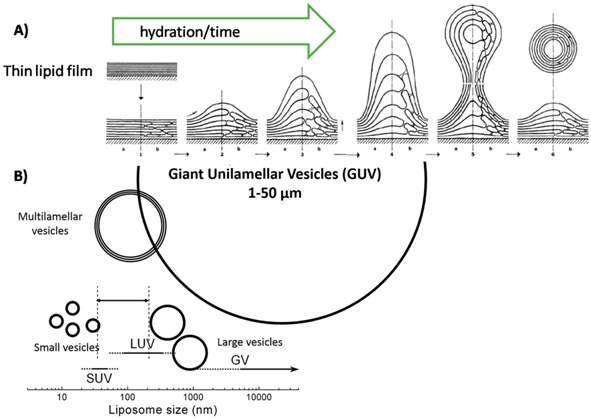

A) Schematic representation for the formation of multilamellar GVs; B) Sizes and types of different GVs, vesicles and other liposome-like objects.

Firstly, the role of the medium in itself should always be considered since its properties, ionic strength for instance [55], largely influence the reactivity and the interactions of the biotic macromolecules. Many kinds of media are relevant for the formation of protocells, such as mineral surfaces [51]. Indeed, they represent supports where organic molecules can be fixed and where their proximity favor polymerization reactions. Thus, non-enzymatic polymerizations of peptides or nucleic acids are obtained from precursors in these conditions [45]. Moreover, various types of minerals, such as montmorillonite, can facilitate membrane assembly from micelles and RNA can even be encapsulated in these conditions [56,57]. As all the components of the protocells can be formed and reunited on mineral surfaces, their study is important to unravel the emergence of biological systems.

Secondly, the role of the interactions between the different components of the protocells should not be underestimated when considering the steps of their formations. It was proven lately that nucleobases, sugars and potentially amino acids, are responsible for the formation and the stability of fatty acid membranes [58]. Once formed, lipid membrane environments can be set up by various compounds. For instance, hydrophobic dipeptide synthesis occurs within the fatty acid membrane and these dipeptides may reinforce, in return, the stability of the lipid bilayer [59]. Small peptides, such as cationic hydrophobic peptides, are also responsible for the recruitment of additional elements around the membranes. Indeed, they can interact at the same time with hydrophobic membrane lipids and anionic oligonucleotides and enable the association between RNA and primitive membranes [60]. Membrane surfaces could then become stabilized by platforms supporting RNA-catalyzed reactions. These different results tend to emphasize the necessity to take into account all the protocell components in a systemic approach to understand how the system can be formed. Indeed, their cooperation is essential for the setting up of the protocell.

## 3. Protocell Metabolism

In order to be functional, a protocell should profit from a metabolism which involves several conditions (Figure 6).

The first condition is the ability to exchange with the medium to get the elements necessary for its functioning and to evacuate the waste [25]. These transfers are regulated by the composition of the membrane. In modern cells, membranes are rather permeable to hydrophobic components and could not be conducive to the exchange of other molecules without proteins [53]. However, in bottom-up approaches, fatty acid membranes are an interesting model for protocells because they are more dynamic than phospholipid membrane, and they allow by simple diffusion of the entry of charged molecules as nucleotides [61]. Other components integrated to the membrane, such as RNA, could also influence the permeability of the membranes [61].

The second condition is a source of energy in order to achieve the various reactions that a protocell needs to perform: Syntheses, transports, replication and waste elimination. Bottom-up approaches try to understand which prebiotic sources of energy could supply protocells, one of them being light energy as a result of intermediaries, such as the Polycyclic Aromatic Hydrocarbons (PAH). They are amphiphilic compounds present on the young Earth which can stabilize fatty acid vesicles, as it is the case for 9-anthracene carboxylic acid in decanoic acid membranes [62]. In lipid bilayers, these aromatic molecules are also at the origin of a photon-induced production of protons within a vesicle [63]. Then, this supply could have provided the metabolism of the first living organisms. Natural and exploitable proton gradients across membranes were also possible in hydrothermal vents since they could have been saturated in H_2_ and exposed to a large pH range (~5–11) [43]. 

The third condition corresponds to catalytic activities as they are mandatory for the functioning and the replication of the protocell. In that case, top-down approaches are favored since catalyzes require efficient enzymes [48]. Lipids synthesis, for instance, was shown to be possible in decanoic acid vesicles, including a ruthenium complex spontaneously added to the membrane. This catalyst is responsible for the hydrolysis of lipid precursors in amphiphilic compounds which are directly inserted in the membrane [64]. Protein expression in synthetic cells has also been studied considerably since its first description in liposomes containing a cell-free transcription, translation system and expressing a mutant green fluorescent protein (GFP) [65]. Recently, a reconstructed system was able to exploit light energy for protein expression. Indeed, the bacteriorhodopsin and the ATP-synthase inserted in a giant vesicle were responsible for the production of ATP from light energy. This energetic compound fueled an encapsulated cell-free protein expression system at the origin of the expression of bacteriorhodopsin and some proteins of the ATP synthase [66].

It is noticeable that a protocell should have a metabolism which can be influenced by sensing the environment as it is the case for modern cells [67]. Indeed, a modification in the medium or a communication between individuals can be at the origin of an adapted response for a cell [68]. Top-down approaches have successfully tried to reproduce that kind of behavior. For instance, some artificial cells could communicate since the presence of theophylline induced an IPTG release by themselves, hence the expression of the *lac* operon in the neighbor bacterium *E. coli* [69]. Recently, a quorum-sensing was reproduced in artificial cells. These cell-mimics possess highly diffusive boundaries allowing to release the transcription factor T3 RNA polymerase as a message in the population. This protein efficiently caused the expression of fused proteins in the other synthetic cells [70]. These approaches underline how a protocell could react to the environment variations and to respond in consequence in order to persist. 

## 4. Replication of the Protocells

A protocell responding to the definition of the artificial life is able to self-replicate [47]. Most of the time, two parts can be distinguished during this step—the reproduction of the content and the reproduction of the container [25] (Figure 6).

### 4.1. Nature and Replication of the Content

The nature of the molecule carrying the protocell information remains elusive and many models have been proposed [51]. Among them, some scenarios emphasize the fact that life emerged from metabolic networks able to self-reproduce [71]. The most detailed one is probably the graded autocatalysis replication domain (GARD) model. This theory relies on the fact that compositional genomes (composomes) could be coded in the lipidic components of a system. This information could be maintained during replication cycles and could predate information based on a sequenced polymer as it is the case in modern cells [72]. Nevertheless, it was shown that the replication of composomes is too imprecise to be efficiently maintained by selection [73]. 

Otherwise, scenarios involving information conserved in sequenced molecules, as it is the case in modern cells, have also been suggested. The RNA-world hypothesis highlighting RNA as the first carrier of information remains the most famous one [26]. This is notably due to the fact that some catalytic RNA, called ribozymes, could at the same time act as information carriers and catalysts for their self-replication and other chemical reactions. Bottom-up approaches based on the RNA-world and the non-enzymatic replication of oligonucleotides are numerous, such as the ones focusing on systems supplemented with citrate. This compound chelates to Mg^2+^ causing its precipitation in salts. Mg^2+^ is a cation which is necessary for ribozymes activity but it tends to disrupt fatty acid membranes. Thus, in the presence of citrate, protocells are protected against the destabilizing effect of Mg^2+^ while they could still profit from its activation effect on ribozymes [74]. Otherwise, it is established now that fatty acid membranes are permeable enough to allow nucleotides diffusion towards the lumen [75]. Lately, citrate chelated to Mg^2+^ was shown to be also responsible for a higher permeability of fatty acid membranes to short oligonucleotides increasing the non-enzymatic copying of RNA templates within the vesicles [76]. These results are bringing research closer to a system in which the information carrier could be reproduced. However, in the absence of a prebiotic pathway for citrate synthesis, other plausible compounds with similar properties should be sought. These studies illustrate that despite major achievements, considerable research is required before the discovery of a system in which a ribonucleotide can be replicated non-enzymatically [77]. Actually, the RNA-world theory involves that a ribozyme could eventually be able to catalyze its self-replication. In one study, an exponential growth of self-replicating ribozymes was observed in a mixture containing a small number of ribozymes, MgCl_2_ and which was periodically supplied with RNA substrates [78]. However, only a limited Darwinian evolution could be observed and studies are far from obtaining a ribozyme which could self-replicate, mutate and evolve [26].

Other hypotheses exist concerning the content of the first living system, such as a combination of small oligonucleotides and short peptides sharing the informative and the catalytic roles [79]. Indeed, even though RNA has high potential as information carriers, it is likely that catalytic ribozymes were too sophisticated to be present in the first living systems [80]. In return, short peptides, such as the dipeptide SerHis, reveal a high potential as primitive organocatalysts despite their simplicity [81]. In this environment, a precocious appearance of translation could have led to a coevolution between peptides and oligonucleotides towards the longer polymers present in modern cells [79]. Indeed, it was proven lately by sequence alignments that aminoacyl tRNA synthetases of both classes I and II originally originate from the same ancestral gene. This result suggests that a translation from codons to amino acids with a simple genetic alphabet was performed by enzymes very early on an evolutionary timescale [82]. Besides, recent works on the population dynamics of the replication of codon-containing genomes show that the necessary transition from an operational RNA-world towards a less evolved translation system is not viable, which again insinuates an early-coevolution between replication and translation [83].

Top-down approaches can also help to address the question of the replication. A major achievement was based on a synthetic cell inspired by the Φ29 virus containing DNA and a set of expression enzymes (PURE system) allowing the expression of the encoded proteins. These proteins were responsible for the DNA replication and the establishment of a system able to catalyze the self-replication of its genetic information [84].

### 4.2. Replication of the Container: Growth and Division of Giant Vesicles

The chemical nature of the first cell compartment is as unseizable as the mechanism of its replication. Membraneless coacervates are good models since they could self-reproduce if the conditions of the medium are transiently modified [25]. However, primitive compartments made of lipids remain the privileged models since they are similar to modern cells [25] and giant vesicles constituted by fatty acids were the first models of protocells [85]. The preparation step of such compartments was exhaustively reviewed elsewhere [86], however, what is a giant vesicle (GV), why and how to prepare it, is briefly discussed in Box 2.

**Figure 7:**
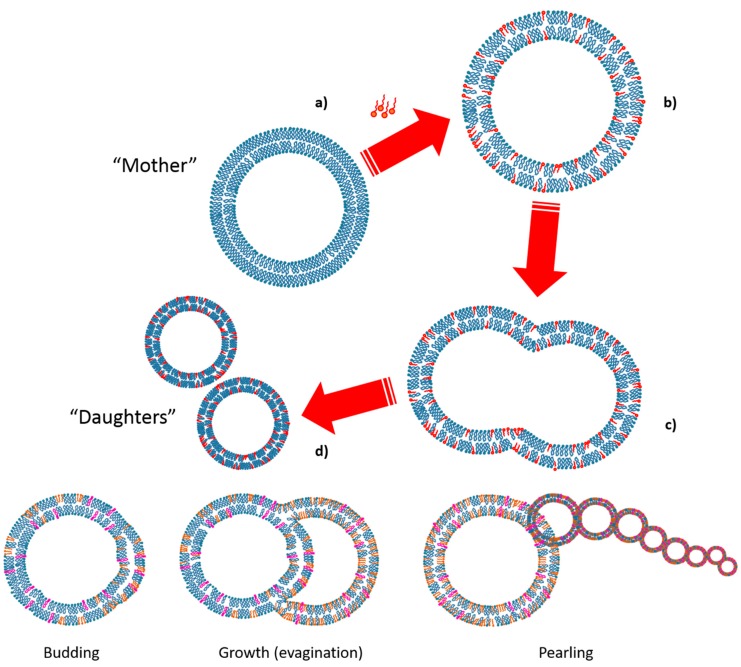
Schematic representation of vesicles self-reproduction. This process, already called growth and division, is a process in which a growing vesicle (**a→b**) first transforms its spherical shape into a dumbbell shape (**b→c**), and then splits into two spherical daughter vesicles (**c→d**). Budding, growth and pearling are some other processes part of the growth and division process, however not always clearly observable.

Any system able to replicate itself can be indicated as an autopoietic system [87]. Lipid vesicles self-reproduction occurs with a growing vesicle taking a non-spherical form and eventually dividing into two or more spherical daughter vesicles (Figure 7). This process is commonly called growth and division (GD) [88,89]. The first experiments carried out to monitor the GD phenomenon were achieved on vesicles formed with the simplest plausible prebiotic amphiphiles able to form bilayer upon hydration at basic pH, the fatty acids [36,90]. In these studies, the processes of GD of several types of giant vesicles were essentially assessed by microscopic observation, and with the increase of vesicle in size and number as a proof of the mechanism [88,91]. For some of these pioneering works, oleic acid vesicles were able to grow and to divide with the supply of oleic acid coming from anhydrides hydrolyzed at alkaline pH by the vesicles themselves [92,93].

Since these findings, several ways to achieve GD have been shown. Among them, the addition of fatty acid micelles to fatty acid vesicles leads to the growth [56,94] and eventually to the division under a slight agitation [95]. During the process of GD, many changes of morphology can be observed for the vesicles, such as the budding (Figure 7) [96]. Another possibility is the pearling which results from the fact that a multilamellar vesicle supplied with amphiphiles possess an external membrane layer which grows faster than the internal ones [95]. Interestingly, in some systems as oleic acid vesicles, the size of the vesicles after growth and division stayed similar. This mechanism is called the matrix effect and would conveniently explain the maintenance of the protocell size through division [88,97,98].

The GD phenomenon has also been studied in the case where the vesicles were filled with biopolymers. Curiously, the presence of a membrane-bound protein, called Zein, induces a contraction of the liposome membrane and the growth with a supply of vesicles [99]. Macromolecules in the lumen of the vesicles also entail the growth because the encapsulation involves an osmotic pressure applied to the membrane which forces the supply of amphiphiles, as it is the case with RNA [100]. However, encapsulated biopolymers could also lead to the division of the liposome as it is the case for PEG 6000 delivered by electrofusion of vesicles which provokes the budding due to the depletion volume effect [101]. Regardless of the approach, the question of the content preservation after the division is still at stake. Recent results tend to show that it is not a random process and that macromolecules, as carbonic anhydrase, could be selectively conserved with replications [102].

Regarding top-down approaches, the chemical synthesis of amphiphiles within a liposome was also performed in many systems in order to achieve the GD [64,103,104,105]. Another striking result was the expression of proteins from DNA with added enzymes (PURE system) responsible for lipid syntheses in a synthetic cell [106]. This production could also lead to protocell division.

Despite the fact that the growth and division of original lipid boundaries was proven, it was not possible to distinguish pre-existing vesicles (mothers) that have grown in size by incorporating amphiphiles (i.e., fatty acids or fatty acids micelles) from vesicles generated by a vesicle division process, nor from de novo formed vesicles (daughters). Among others, a few relevant experimental setups have been reported. Firstly, a detection cargo, ferritin, was included into mother vesicles, that was distributed during division between daughter vesicles [107,108,109]. Secondly, non-exchanging (well anchored) FRET (Fluorescence Resonance Energy Transfer) probes were incorporated into original fatty acid vesicles [56,94,95,110,111,112,113]. However, these approaches, did not allow an independent characterization of the lipid composition, size or dimensions of each aggregate type.

**Figure 8:**
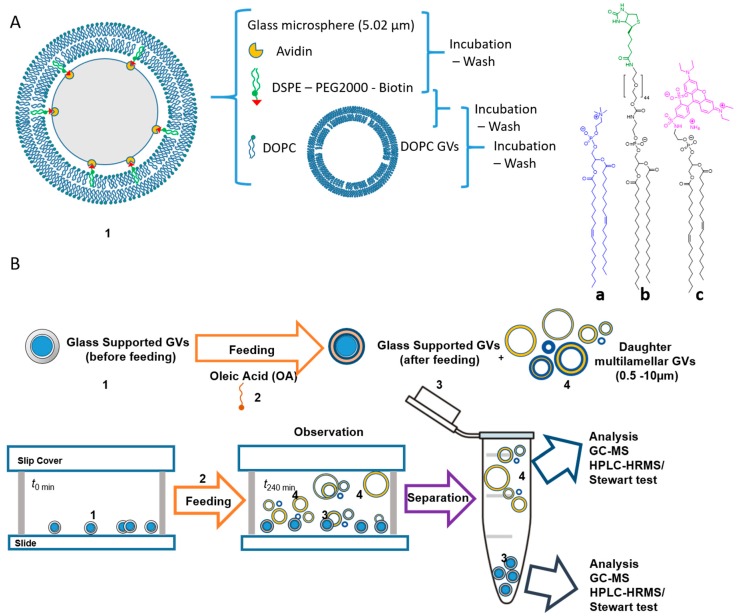
Preparation and use of DOPC SGVs (**1**) for the monitoring and analysis of the growth and division processes. (**A**) 1,2-dioleoyl-*sn*-glycero-3-phosphocholine (DOPC, **a**), was anchored by first treating glass beads with avidin/biotin-DSPE (1,2-distearoyl-*sn*-glycero-3-phosphoethanolamine-N [biotinyl(polyethylene glycol)-2000], (**b**), then adding multilamellar DOPC vesicles from which a bilayer was transferred to coat the beads; a fluorescent lipidic probe the DOPE-Rh^+^ (1,2-dioleoyl-*sn*-glycero-3-phosphoethanolamine-N-(lissamine rhodamine B sulphonyl) ammonium salt (**c**). (**B**) A supported DOPC giant vesicles (**1**, SGVs, grey circle filled blue) fed with oleic acid (OA, 2, orange) grow through incorporation of OA and divide into supported GVs (**3**, orange circles filled blue) and new unsupported vesicles (**4**, blue rings around yellow circles). The composition of the membranes of SGVs (**3**) and daughter GVs (**4**) was elucidated by chromatographic analysis associated with mass spectrometry or by the Stewart test [115].

A solution to this problem was given by the use of fatty acids or phospholipid vesicles that were supported by monodispersed glass beads (5.02 µm). These tools are also named microsphere-supported giant vesicles (SGVs). The size of the SGVs classifies them as giant vesicles, which can be easily prepared and with a composition which could be, in principle, the same complex as the one of a cell. The SGVs are an effective tool for they allow a separation by simple centrifugation between the mother vesicles from the daughters after GD (Figure 8) and their subsequent analysis. Besides, these supported structures present the same environment to external amphiphilic material as any unsupported system. Monnard and colleagues were the first to use decanoic acid SGVs to monitor the transmission of a catalytic function: A ruthenium complex that acts both as a photosensitizer and a redox catalyst, during a replication (i.e. the GD process) [64]. Furthermore, SGVs were prepared in order to unambiguously separate the mother from the daughter vesicles in a GD system after a feeding process. This allowed the analysis of the content of phospholipids and fatty acids for these two populations, and to study the transmission of physico-chemical characteristics from mother to daughter vesicles [114].

Finally, a functional protocell should achieve the replications of the content and the container together in a core-and-shell reproduction [88]. Thus far, a small number of results have been obtained concerning this perspective and they only result from top-down approaches. One example is a system in which the DNA contained in giant vesicles is replicated with PCR reagents during thermal cycles. After that, the lipid production was achieved by amphiphilic catalysts from lipid precursors leading to the growth of the membrane and the division of the giant vesicles [103].

## 5. Protocell Evolution

A functional protocell that is able to self-replicate has the potential to proliferate. However, it can enter in competition with other chemical systems. In that case, the protocell with the highest fitness should prevail on the other. It could lead to the evolution of the protocells if a selection process is applied (Figure 6).

**Figure 9:**
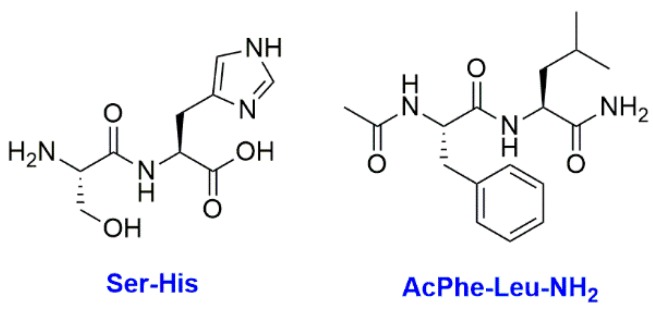
The structure of the catalytic dipeptide SerHis and the membranogenic dipeptide AcPheLeuNH_2_.

Synergies between the different components could be at the origin of a higher fitness, meaning a higher replication potential. One simple cooperation exists between fatty acid vesicle growth and its membrane composition. Indeed, in a vesicle, the dipeptide SerHis catalyzes the formation of a membranogenic dipeptide AcPheLeuNH_2_ (Figure 9) that inserts into the membrane where it is highly affine for fatty acids. This interaction stabilizes the membrane and then the growth of the vesicle is facilitated. Thus, this vesicle is more prone to grow and to proliferate [116]. Another example of synergy is the link between fatty acid vesicle growth and the activity of the contained ribozymes. Some ribozymes can be properly folded and activated only if they are encapsulated [117]. Moreover, some ribozymes need to stay at a low concentration to remain active in order to avoid a high concentration of oligonucleotides which can be inhibitors [118]. Thus, if the concentration of ribozymes is too high in a vesicle their activity could be impaired. This issue could actually be spontaneously solved by the fact that a high concentration of RNA involves a high osmotic pressure and a membrane tension, which is released by an intake of fatty acids and the growth of the vesicle [100]. If the ribozymes were able to self-replicate, then the cycle would indefinitely continue, leading to membrane growth and eventually to division. This kind of network based on cooperation could be at the origin of an increased fitness for the system. It would then be more prone to win the competition against other protocells and to be conserved if a selection process is applied.

Selection is one of the key steps to explain how a system can evolve. Indeed, evolution requires replication, inheritable variation and eventually selection among variants [119]. Experimental evolution gained considerable interest lately in order to understand the minimal systems which can evolve [120]. The lipid composition change in the membranes is an interesting hypothesis for evolution. The first protocell membranes are supposed to be made of fatty acids which are abundant on the primitive Earth [5]. This is a convenient model because fatty acid membranes are intrinsically highly permeable and dynamic without transmembrane proteins [61]. However modern membranes are mainly constituted by phospholipids and so a transition would have occurred from primitive membranes. From this scenario, protocells competing for fatty acids grow faster if they synthesize phospholipids from fatty acids [111]. Furthermore, hybrid bilayers containing both fatty acids and phospholipids get, at the same time, the properties of permeability given by the former and the stabilization given by the latter [113]. Thus, the stability to high Mg^2+^ concentrations of these vesicles make them protocells in which enzymatic and non-enzymatic replication of RNA but also transcription and translation could occur [113]. From these protocells, the emergence of proteins functioning as transporters and fluidity enhancers could explain the transition towards modern membranes.

The evolution of protocells should also be considered within a population since the interactions between the individuals can also shape their own future. In that regard, a study, among others, taking a top-down approach was performed with a population of protocells gathering protease-containing coacervates and proteinosomes containing single-stranded DNA. The interaction between a predator (a coacervate) and a prey (a proteinosome) induced the hydrolysis of the latter and the uptake of DNA in the killer protocell. This trafficking was responsible for the ability for the predator to kill again [121]. This kind of network highlights the necessity to take into account the communications and the relationships between several kinds of interacting systems to understand the evolution for each of them. 

## 6. Concluding Remarks

Previously, this study emphasized the importance of considering protocells as systems constituted of a wide variety of compounds. Indeed, this diversity is at the origins of complex networks based on physical and chemical interactions from which synergies can emerge [2]. The cooperations between these molecules are of paramount importance for protocells as they are involved in their formation, their metabolism, their replication and finally their evolution. However, this approach of systems chemistry is facing two major hurdles in the origins of life study which need to be overridden in order to accelerate the progress.

The first impediment is a technical one. As no prediction could be finalized, at first glance, for the appearance of synergies, many systems should be tested. Indeed, they can differ for the number of tested molecules, their natures or even their combinations. This represents a real challenge to analyze a large volume of media, and overall, to study this complex media. In order to understand it, new tools have been used recently [2]. Notably, the microfluidic and nanofluidic devices provided the opportunity to investigate numerous and controlled media [120] and the dynamic combinatorial chemistry (DCC) aimed to examine how a network of molecules reacts [2]. They are particularly well suited for the origins of life study as they allow to reproduce prebiotic conditions (the composition but also the physical parameters) and to observe the patterns that could emerge. Taking into account the complexity of the media studied, new techniques based on theoretical simulations to investigate reactivity and interactions could be of great assistance to gain a better understanding about the origins of life.

The second obstacle is a problem of knowledge. Indeed, prebiotic environments cannot be perfectly described as they are not available anymore. Therefore, it remains a hard task to understand which environment could be suitable for the origins of life. Fortunately, geological and chemical studies are still focusing on these aspects to find the conditions associated to prebiotic environments [122]. Among all the different proposed environments, hydrothermal fields [44] and hydrothermal vents [43] are the most conducive. However, vesicles can assemble from amphiphilic compounds in hydrothermal hot springs, while seawater tends to destabilize the membranes due to the high concentration of ions [123]. Moreover, short polymers are formed in hydrothermal vents, but they are subsequently hydrolyzed in these environments where a population of polymers cannot withdraw from equilibrium. Hydrothermal fields, on the contrary, are conducive for the formation of polymers longer than 50 base units and which could plausibly have a catalytic activity [124]. In order to unravel the mystery of the origins of life, it is clear that such studies based on simulated prebiotic environments should be favored to be as realistic as possible.

## Figures and Tables

**Table 1:** **Proteogenic** and non proteogenic aminocids found in Miller’s and Miller’s—Bada revised experiments.

Remarks	1953 & 1955 Miller’s/2003 & 2014 Bada revised experiments	
**Found in all the experiments**	**Glycine** (Gly) ^a,b,c^, **α-Alanine** (Ala) ^a,b,c^, β-Alanine, **Aspartic acid** (Asp) ^a^, α-Aminobutyric acid	
Additional molecules found in 2003 and 2014 experiments	**Serine** (Ser) ^a,c^, Isoserine, α-Aminoisobutyric acid, β-Aminoisobutyric acid, β-Aminobutyric acid, γ-Aminobutyric acid, **Valine** (Val) ^a,c^, Isovaline, **Glutamic acid** (Glu) ^a^	
Additional natural and non-natural amino acids found only in the experiments carried out in 2003 and 2014	**2003**	**2014**
	Norvaline, α-Aminoadipic acid, Homoserine, 2-Methylserine, β-Hydroxyaspartic acid, Ornithine, 2-Methylglutamic acid, **Phenylalanine** (Phe) ^a^	Homocysteic acid, S-methylcysteine, Methionine (Met) ^a^, **Methionine** sulfoxide, Methionine sulfone, Isoleucine (Ile) ^a^, **Leucine** (Leu) ^a,b^, Ethionine

^a^ proteogenic aminoacids found; ^b^ aminoacids that can be obtained directly from formamide accordingto Saladino and colleagues (Figure 1); ^c^ aminoacids could be obtained via the intermediaries proposed by Sutherland and colleagues (Figure 2).
